# Evaluation of a Community Pharmacist-Led Intervention Program for Early Detection of Gastrointestinal Adverse Events of Dipeptidyl Peptidase-4 Inhibitors: A Multicenter, Non-Randomized Comparative Study

**DOI:** 10.3390/pharmacy13050119

**Published:** 2025-08-28

**Authors:** Ayana Funabashi, Hitoshi Ito, Mamoru Maeda, Yoshitaka Hasegawa, Ryota Tsukioka, Mitsuko Onda

**Affiliations:** 1 AIN PHARMACIEZ INCORPORATED 1F, 2-7-1, Daigakumachi, Takatsuki 569-8686, Osaka, Japan; ayana.funabashi.iw2@ainj.co.jp; 2 Faculty of Pharmacy, Department of Social and Administrative Pharmacy, Osaka Medical and Pharmaceutical University, 4-20-1, Nasahara, Takatsuki 569-1094, Osaka, Japan; 3 AIN HOLDINGS INCORPORATED JR South Shinjuku Building 11F, 2-1-5 Yoyogi, Shibuya, Tokyo 151-0053, Japan; hitoshi.ito.es4@ainj.co.jp (H.I.); mamoru.maeda.36p@ainj.co.jp (M.M.); yoshitaka.hasegawa.x9z@ainj.co.jp (Y.H.); ryota.tsukioka.k1t@ainj.co.jp (R.T.)

**Keywords:** dipeptidyl peptidase-4 inhibitors, diabetes mellitus, gastrointestinal adverse events, follow-up care, tracing report, community pharmacist

## Abstract

In this multicenter, nonrandomised comparative study, we evaluated the potential effectiveness of a program to promote the safe use of dipeptidyl peptidase-4 (DPP-4) inhibitors led by community pharmacists. The program facilitated early detection of gastrointestinal adverse events (GIAEs) in patients newly prescribed DPP-4 inhibitors and facilitated timely communication with physicians. Community pharmacists reviewed patient conditions and provided relevant information to physicians as needed. GIAE monitoring based on the program was conducted in 35 patients at 10 pharmacies in Japan (intervention group) between March and August 2024. The proportion of pharmacist interventions was compared with that in 451 patients from March to August 2023, before program implementation (baseline cohort). The primary outcome, pharmacist intervention rate, was significantly higher in the intervention group (5 out of 35 patients, 14.3%) than in the baseline cohort (0 out of 451 patients, 0.0%) (*p* < 0.001). GIAEs were identified in 13 out of 35 patients (37.1%) in the intervention group; information for five patients (14.3%) was shared with physicians, resulting in discontinuation of the DPP-4 inhibitor in one patient and addition of supportive therapy in others. Most GIAEs occurred within the first 1–2 weeks of therapy, highlighting the need for early intervention. Thus, proactive involvement of community pharmacists may improve the care process in these cases and contribute to healthcare coordination and diabetes care quality.

## 1. Introduction

Dipeptidyl peptidase-4 (DPP-4) inhibitors lower blood glucose levels by increasing the activity of the incretins glucose-dependent insulinotropic polypeptide (GIP) and glucagon-like peptide-1 (GLP-1), thus inducing insulin secretion in a glucose-dependent manner [1]. Meta-analyses have shown that DPP-4 inhibitors are particularly effective in Asian patients [2]. Furthermore, a previous study utilizing the Japanese National Insurance Claims Database found that DPP-4 inhibitors were the most commonly prescribed first-line treatments for Japanese patients with type 2 diabetes [3]. However, incretins also exert extrapancreatic effects that delay the evacuation of gastric contents [4,5,6]. Gastrointestinal adverse events (GIAEs) such as nausea, vomiting, decreased appetite, constipation, and diarrhea have been reported in previous studies and post-marketing surveillance [7]. Additionally, GIAEs may be indicators of the onset of more serious adverse effects, such as acute pancreatitis or intestinal obstruction, which were associated with DPP-4 inhibitor use [8,9].

Community pharmacists in Japan are primarily responsible for the pharmacological management of outpatient drug therapies. They supply patients with information on the effects, proper use, precautions, side effects, and interactions of prescribed medications as specified in the physician’s prescription. Community pharmacists also provide follow-up care after dispensing medications through telephone calls or other communication methods where necessary [10,11,12]. This entails monitoring the condition of the patient during drug therapy, including medication adherence, use of other drugs (including over-the-counter products), the occurrence of adverse drug reactions, and the patient’s living environment. Pharmacists are expected to respond appropriately when issues are identified [12]. Community pharmacists may document and report to physicians any patient information deemed relevant for follow-up care, using a “tracing report” [13,14]. Several previous studies have shown that providing follow-up care and sharing information with physicians through tracing reports aids in optimizing drug therapy [15,16]. Tracing reports are written documents that community pharmacists use to provide feedback to physicians about patient information. While this information may not be urgent, it is important for appropriate clinical decision-making [14].

Given this background, community pharmacists should actively engage in follow-up care for patients prescribed DPP-4 inhibitors to treat type 2 diabetes. This is essential to detect and address GIAE early, preserving patients’ quality of life, and ensuring safe and effective medication use. It is desirable to establish appropriate timing and methods for follow-up care to enable community pharmacists to early detect these symptoms The Japanese Pharmacists Association has established and published basic guidelines for follow up care [12]; however, these guidelines lack practical details on when to follow-up, how to share patient information with physicians, and effective communication approaches. Additionally, few studies have examined the content and effectiveness of follow-up care provided by community pharmacists. Therefore, in this study, we aimed to develop a community pharmacist-led intervention program and evaluate its effect on the early detection and management of GIAEs in patients using DPP-4 inhibitors.

## 2. Materials and Methods

### 2.1. Study Design

This study was designed as a multicenter, non-randomized comparative study.

### 2.2. Study Setting

The study sites were selected from pharmacies in the Kansai region operated by a pharmacy chain based in Japan, specifically those ranked in the top ten in terms of the number of prescriptions containing DPP-4 inhibitors.

### 2.3. Inclusion and Exclusion Criteria

Patients who presented at one of the ten participating pharmacies and received dispensed medications for type 2 diabetes were included if they met the following eligibility criteria (the same criteria were used for the baseline cohort):

- The patients had not been prescribed any DPP-4 inhibitors approved and marketed in Japan (sitagliptin, linagliptin, teneligliptin, vildagliptin, alogliptin, saxagliptin, anagliptin, omarigliptin, or trelagliptin) within the past year and were newly initiated on one of these agents.
- Patients provided written informed consent to participate in the study.

Patients who met any of the following exclusion criteria were excluded from the study:

- Current use of antipsychotics, antidepressants, or corticosteroid hormones;
- Under 18 years of age;
- Did not provide written informed consent to participate in the study;
- Deemed by the responsible pharmacist to be unsuitable for participation;
- Unlikely to be continuously followed up for 6 months because of relocation or other reasons at the time of enrolment.

### 2.4. Intervention

**Figure 1:**
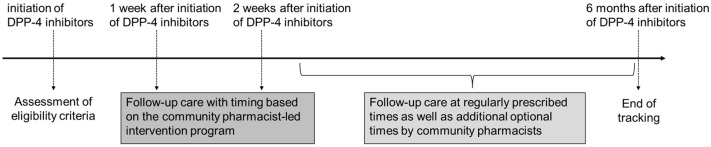
Timeline of the Community Pharmacist-Led Intervention Program for Monitoring Gastrointestinal Adverse Events After Initiating Dipeptidyl Peptidase-4 Inhibitor Therapy. Each patient was monitored for 6 months after initiating DPP-4 inhibitor therapy, as shown in the timeline. DPP-4 inhibitors: dipeptidyl peptidase-4 inhibitors. Follow-up care: assessment of clinical status and medication adherence during the treatment period, with pharmacist-led interventions in response to identified issues. Community pharmacist-led intervention program: a structured protocol defining the timing of follow-up care, procedures for sharing patient information with physicians, and methods for interprofessional communication.

We defined “intervention” as the process in which pharmacists identified GIAEs in patients who had been newly prescribed DPP-4 inhibitors and subsequently reported these events to the relevant physicians. The study period spanned from March 2024 to February 2025. Community pharmacists conducted follow-up care for eligible patients for six months from the beginning of DPP-4 inhibitor therapy (Figure 1). Based on the community pharmacist-led intervention program, scheduled and structured follow-up care was conducted, typically 1 to 2 weeks after treatment commenced (Pattern A). However, pharmacists sometimes determined that follow-up beyond the scheduled time points was necessary depending on the patient’s condition. Therefore, with the predefined schedule, follow-up care was also provided during patients’ regular visits to the pharmacy for prescription refills (Pattern B), and at unscheduled times based on the pharmacist’s clinical judgment (Pattern C). Pattern B was limited to regular visits, while Pattern C had all other intervention timings outside of those visits, with no overlap between these patterns. The presence or absence of GIAEs was confirmed during all types of follow-up. If community pharmacists identified a GIAE, they provided physicians with a “tracing report” containing detailed information on the specific symptoms, timing of onset, and course of exacerbation or remission, as necessary.

### 2.5. Supplementary Information on the Community Pharmacist-Led Intervention Program

#### 2.5.1. Timing of Follow-Up Care

Using the Japanese Adverse Drug Event Report database (JADER), which compiles adverse drug reaction reports in Japan, we analyzed trends in the reporting of DPP-4 inhibitor-related GIAEs (Supplementary Table S1). Based on the study results, follow-up care was provided for the most frequently reported GIAEs (nausea and vomiting, loss of appetite, constipation, and diarrhea). Considering the interquartile range that includes 75% of the onset timing, it was important to initiate follow-up care as early as possible from the first quartile values for gastrointestinal symptoms (nausea and vomiting: 3.0 days; diarrhea: 5.0 days; constipation: 21.0 days; loss of appetite: 15.0 days), as shown in Supplementary Table S1. Consequently, the timing of follow-up care was determined to be appropriate at 1 to 2 weeks after starting DPP-4 inhibitor therapy. However, when community pharmacists conduct follow-up care outside of the patient’s regular pharmacy visits, they must first schedule an appointment by contacting the patient in advance. Therefore, if the timing of follow-up is strictly fixed, there is a risk that the intervention may not take place owing to scheduling conflicts on the patient’s side. To address this concern, the timing of the intervention was set flexibly, allowing it to occur either one or two weeks after treatment initiation. Additional follow-ups were performed during patient visits to the pharmacy and whenever deemed necessary by the community pharmacist.

#### 2.5.2. Follow-Up Care Implementation Form

For community pharmacists to provide follow-up care to participants, a checklist of items was compiled, and an implementation sheet was developed (Supplementary Figure S1). Before study initiation, community pharmacists underwent a training session at the study sites to ensure uniform understanding of the follow-up care protocol and proper use of the implementation sheet.

### 2.6. Establishment of the Baseline Cohort

Before implementing community pharmacist-led intervention program, a baseline cohort was established at ten selected pharmacies to obtain comparative data. Patients who were newly prescribed DPP-4 inhibitors between March and August 2023 were included in the baseline cohort. The intervention status was evaluated by examining the content of the “tracing reports” submitted to physicians by community pharmacists after providing follow-up care during the first six months of DPP-4 inhibitor treatment, along with the patients’ progress records.

### 2.7. Clinical Outcomes

#### 2.7.1. Primary Endpoint

Percentage of patients whose DPP-4 inhibitor use was reduced or discontinued because of GIAEs

#### 2.7.2. Secondary Endpoints

Proportion of patients with a confirmed improvement in GIAEs

- Percentage of patients who received community pharmacist-led intervention.

### 2.8. Statistical Analysis

To evaluate clinical outcomes, we calculated the following proportions in the intervention group: (1) the percentage of patients whose DPP-4 inhibitor therapy was reduced or discontinued owing to gastrointestinal adverse events (GIAEs) (primary outcome), (2) the percentage of patients who confirmed an improvement in GIAEs following the intervention (secondary outcome), and (3) the percentage of patients for whom pharmacists identified GIAEs after newly initiating DPP-4 inhibitors and subsequently reported these events to physicians (secondary outcome). For comparison, the same proportions were calculated for patients newly prescribed DPP-4 inhibitors between March and August 2023 in the baseline group. Fisher’s exact test was used to compare the proportions before and after the intervention, with the significance level set at 5%.

GIAEs were categorized into three patterns (Patterns A, B, and C) based on follow-up timing, as previously defined. For each pattern, we documented the presence and number of GIAEs classified by symptoms, along with a descriptive analysis of their content and outcomes. In this study, nausea and vomiting, loss of appetite, constipation, and diarrhea, which have been reported as common GIAEs in previous surveys on DPP-4 inhibitors, were identified as the main GIAEs. Additional GIAEs were aggregated as “Other GIAEs” if detected. When multiple GIAEs were identified in a single patient, each was counted separately, and duplicates were allowed.

## 3. Results

### 3.1. Patient Characteristics

In the baseline cohort, 451 patients were included. During the intervention period, 36 patients met the eligibility criteria and agreed to participate (Table 1). Among these, 35 were included in the intervention group, while one was excluded based on the exclusion criteria. This indicates that some eligible patients may have declined to participate or were unable to enroll for other reasons.

### 3.2. Comparison of Outcomes

In the intervention group of 35 patients, community pharmacists identified GIAEs during follow-up care in 13 patients (37.1%). Among these, five patients (14.3%) received follow-up care, had GIAEs identified, and had information shared with physicians through tracing reports. This was significantly higher than in the baseline cohort (0.0%) (*p* < 0.001). Additionally, in the intervention group, GIAEs led to discontinuation of DPP-4 inhibitors in one patient, while improvement of symptoms was observed in another (Table 2). Notably, tracing reports contain personal information, so patient consent is required before sharing them with physicians. In cases where symptoms were mild and a physician visit was already scheduled shortly after the follow-up care, pharmacists sometimes opted to encourage patients to consult their physician directly instead of sending a tracing report. Additionally, some patients preferred to report their symptoms themselves and did not consent to sharing information via tracing reports. Consequently, tracing reports were submitted for only 5 of the 13 patients.

### 3.3. Evaluation of GIAE Detection

Follow-up care was provided to all 35 patients. The median interval between the initiation of DPP-4 inhibitor administration and the first follow-up was 7 days (interquartile range [IQR]: 7–12 days). Table 3 showed that gastrointestinal adverse events (GIAEs) were confirmed in 13 patients, with Pattern A being the most common in six patients (46.2%). During the observation period, DPP-4 inhibitors were discontinued in three of these 13 patients. Overall, 23 GIAEs were identified among the 13 patients (Table 4). Pattern A was the most frequent with 10 cases (43.5%), followed by Pattern C with 8 cases (34.8%), and Pattern B with 5 cases (21.7%). Constipation was the most common symptom with 11 cases (47.8%), followed by diarrhea with 6 cases (26.1%). The median time from initiation of DPP-4 inhibitor administration to the first symptom confirmation was 18 days (IQR: 11.0–85.0) for constipation and 59.5 days (IQR: 8.5–78.5) for diarrhea.

### 3.4. Interventions by Community Pharmacists After GIAE Detection

Community pharmacists used tracing reports to provide physicians with information regarding five of the 13 patients with confirmed GIAEs. Table 5 presents the patient outcomes. For example, Patient No. 4 developed constipation and gastrointestinal upset and independently discontinued the drug. Patient No. 5 experienced soft stools and constipation, and after consulting her physician, was prescribed probiotics that alleviated her symptoms.

## 4. Discussion

This study suggests that an active intervention program led by community pharmacists for patients newly prescribed with DPP-4 inhibitors may facilitate the early detection of GIAEs. This is a new finding that was not confirmed in the baseline cohort.

This study suggests that an active intervention program led by community pharmacists for patients newly prescribed DPP-4 inhibitors may facilitate the early detection of GIAEs. This is a new finding that was not confirmed in the baseline cohort. Pharmacist-led interventions in diabetes care have been shown in numerous studies to improve HbA1c levels and medication adherence [17,18,19,20]. Furthermore, the Pharmacists’ Patient Care Process established by the Joint Commission of Pharmacy Practitioners in the United States and the follow-up care guidelines from the Japanese Pharmacists Association highlight the importance of pharmacists continuously monitoring adverse events and collaborating with physicians as necessary [12,21]. In this study, we aimed to facilitate the early detection of adverse events through pharmacist intervention by examining the timing of adverse events derived from the JADER database. In this study, we focused on gastrointestinal symptoms associated with DPP-4 inhibitors to evaluate the effectiveness of pharmacist intervention. This study aligns with previous reports evaluating the effectiveness of pharmacist intervention and is expected to offer new insights.

GIAEs, such as nausea, have been reported to be associated with decreased quality of life in patients with diabetes [22,23]. Therefore, early detection and treatment of these side effects are important to ensure the continuity of diabetes treatment. Conversely, previous reports have indicated that most GIAEs associated with DPP-4 inhibitor use are mild and typically do not require treatment interruption [7,24,25]. Consequently, patients may underestimate the clinical significance of these symptoms and be less inclined to report them to healthcare providers. In this study, GIAEs were observed in 37% of patients newly prescribed DPP-4 inhibitors, with pharmacist intervention occurring in 14.3% of these cases. Cautious interpretation is required owing to limitations such as sample size; however, the incidence of confirmed GIAEs was higher than what was reported in post-marketing surveillance data [7]. This suggests that proactive follow-up by pharmacists may have helped to detect mild symptoms that could easily be overlooked in routine clinical practice. Notably, most GIAEs were detected within 1 to 2 weeks after treatment commencement (Pattern A was observed in 46.2% of cases, as shown in Table 3). This aligns with trends observed in the JADER database. Additionally, among the 13 patients with confirmed GIAE, three stopped DPP-4 inhibitor treatment, and one case stopped medication owing to the onset of a rash. This suggests that pharmacist-led follow-up may be essential in preventing the worsening of gastrointestinal symptoms and the early detection of adverse events beyond the gastrointestinal tract. Such timely interventions alleviate patient discomfort and may help prevent more severe complications, including pancreatitis [8,9,26], which are of concern in association with DPP-4 inhibitors. These findings show that a planned pharmacist intervention program focused on the important period after treatment initiation, based on pharmacovigilance data such as JADER, could enhance the quality of pharmacological management and promote safer diabetes treatment.

Constipation was the GIAE most commonly identified through the community pharmacist-led intervention program. This trend aligns with post-marketing surveillance data on sitagliptin in Japan [7]; however, the median onset of constipation was 18 days (IQR: 11.0–85.0) which is earlier than that recorded in the JADER database (Supplementary Table S1). JADER is a spontaneous reporting database that records a large number of serious adverse events [27,28]. Therefore, GIAEs occurring during the pre-symptomatic stage may not be accurately represented in the database. Conversely, six (54.5%) of the 11 cases of constipation identified in this study were detected 1–2 weeks after initiating DPP-4 inhibitor therapy. This suggests that early intervention by community pharmacists may have contributed to the relatively earlier detection of GIAEs, which could explain the difference in the median onset date of constipation compared with JADER data.

The tracing reports were submitted to physicians based on a community pharmacist-led intervention program. These reports included cases in which DPP-4 inhibitors were discontinued as a result of physician decision as well as cases in which patients discontinued the medication independently. In some instances, the patients were prescribed a bowel preparation, which led to symptom improvement. These findings suggest that continuous follow-ups by pharmacy staff may assist physicians in making appropriate clinical decisions, thereby supporting optimal and safe diabetes treatment.

This study has some limitations that should be considered. First, there are limitations associated with the study design and internal validity. This was a non-randomized, non-blinded observational study, and potential confounding factors between the intervention and baseline cohorts cannot be completely ruled out. The intervention group was significantly younger, and concomitant use of medications, including metformin, may have influenced the incidence of GIAEs. Unfortunately, detailed information on other baseline characteristics (existing gastrointestinal conditions, use of other diabetes medications) was not systematically obtained, limiting our ability to assess their potential impact. Furthermore, participating community pharmacists received training before the study to standardize the program’s implementation. However, awareness of being observed as part of the study may have increased the pharmacists’ vigilance and affected their behavior (Hawthorne effect) [29].

Second, there are limitations associated with data sources, measurement bias, and statistical power. The intervention and baseline cohorts were observed during diverse periods, which may have caused bias owing to changes in prescribing practices, counseling behavior, or documentation by community pharmacists. The control data depended on retrospective records, and community pharmacists were not required to report adverse events during that period, potentially resulting in underestimation of baseline GIAEs and informal pharmacist interventions. Therefore, the observed difference in GIAE incidence (37% vs. 0%) may not show an absolute difference in risk but rather reflect increased detection owing to active monitoring. The small sample size of the intervention group (n = 35) also limited statistical power; post hoc analysis showed a power of approximately 26.4%, lower than the commonly accepted threshold of 80%. Moreover, no multivariate adjustment was conducted owing to the limited number of events, and the substantial imbalance in sample sizes between groups may have influenced the robustness of comparisons between groups.

Finally, there are limitations regarding the scope of outcomes and the generalizability of the findings. In this study, we focused primarily on detecting GIAEs, and outcomes such as medication adherence and glycemic control were obtained as supplementary information without standardized measurements. To more comprehensively evaluate clinical impact, future studies should include these endpoints. Furthermore, this study was conducted within a single pharmacy chain in the Kansai region of Japan, where community pharmacists are expected to provide follow-up care using national practice standards [10,11,12]. Since the scope of pharmacy practice may vary internationally, caution is required when generalizing these findings. However, the favorable outcome observed in this study, including the early detection of GIAEs, may offer a conceptual reference for other regions and other pharmacist-led intervention programs.

Considering these limitations, this study’s results show provisional efficacy. Further studies using randomized controlled trials or concurrent baseline cohorts are required to verify the true efficacy. Nevertheless, this study is the first to show that community pharmacists identified GIAEs in patients newly prescribed DPP-4 inhibitors, intervened promptly using a community pharmacist-led intervention program, and evaluated the outcomes. These results may help develop drug therapy management strategies in community pharmacy settings. Since the intervention across multiple pharmacies following standardized training was feasible, the pharmacy chain plans to continue and potentially expand this program. Furthermore, there are also plans to implement systems to continuously evaluate its effectiveness and assess its impact on interprofessional communication.

## 5. Conclusions

Taken together, this study showed that the early detection and management of GIAEs in patients newly prescribed DPP-4 inhibitors were effectively facilitated by active and timely interventions from community pharmacists using a community pharmacist-led intervention program. Furthermore, this study also showed that continuous follow-ups enabled the provision of appropriate feedback to physicians and supported coordinated patient care.

## Figures and Tables

**Table 1:** Patient characteristics.

	Baseline Cohort (n = 451)	Intervention Group (n = 35)	*p*-Value
Sex			0.732 ^b^
Male	271 (60.1%)	20 (57.1%)	
Female	180 (39.9%)	15 (42.9%)	
Age, Mean (SD)	69.0 (14.0)	62.6 (17.2)	0.039 ^c^
Type of DPP-4 inhibitor prescribed ^a^			0.262 ^d^
Sitagliptin	219 (48.6%)	20 (57.1%)	
Linagliptin	127 (28.2%)	9 (25.7%)	
Teneligliptin	50 (11.1%)	4 (11.4%)	
Vildagliptin	23 (5.1%)	0 (0.0%)	
Alogliptin	18 (4.0%)	0 (0.0%)	
Saxagliptin	9 (2.0%)	0 (0.0%)	
Anagliptin	4 (0.9%)	2 (5.7%)	
Omarigliptin	1 (0.2%)	0 (0.0%)	

SD: standard deviation; DPP-4 Inhibitors: dipeptidyl peptidase-4 inhibitors. ^a^ Trelagliptin was not prescribed in either the baseline cohort or the intervention group. ^b^ Calculated using the chi-square test. ^c^ Calculated using Welch’s *t*-test. ^d^ Calculated using Fisher’s exact test.

**Table 2:** Outcomes comparison.

	Baseline Cohort (n = 451)	Intervention Group (n = 35)	*p*-Value
Intervention by community pharmacists ^a^	0 (0.0%)	5 (14.3%)	<0.001 ^b^
Dose reduction or discontinuation of DPP-4 inhibitors due to GIAEs	0 (0.0%)	1 (2.9%)	0.072 ^b^
Improvement in GIAEs after intervention	0 (0.0%)	1 (2.9%)	0.072 ^b^

DPP-4 Inhibitors: dipeptidyl peptidase-4 inhibitors; GIAEs: gastrointestinal adverse events. ^a^ Indicates the number of patients, among those for whom community pharmacists conducted follow-up care and identified GIAEs, whose information was shared with physicians via tracing reports. ^b^ Calculated using Fisher’s exact test.

**Table 3:** Number of patients in the intervention group by GIAE monitoring follow-up pattern (n = 13 patients with GIAEs).

Pattern	Number of Patients (%)
Pattern A	6 (46.2%)
Pattern B	3 (23.1%)
Pattern C	4 (30.8%)

The patterns are defined as follows. Pattern A: Follow-up was conducted 1 and 2 weeks after the initiation of DPP-4 inhibitors, as specified in the community pharmacist-led intervention program; Pattern B: Follow-up was conducted during the patients’ regular pharmacy visits; Pattern C: Follow-up was conducted voluntarily by community pharmacists at times outside those specified in the community pharmacist-led intervention program.

**Table 4:** Timing of GIAEs onset and follow-up pattern distribution in the intervention group (13 patients with GIAEs).

Symptoms	Median Days (IQR) ^a^	Number of GIAEs ^b^			
		Total	Pattern A	Pattern B	Pattern C
Constipation	18.0 (8.5–78.5)	11	6 (54.5%)	2 (18.2%)	3 (27.3%)
Diarrhea	59.5 (31.5–88.3)	6	1 (16.7%)	1 (16.7%)	4 (66.7%)
Nausea and Vomiting	—	1	0 (0.0%)	0 (0.0%)	1 (100.0%)
Loss of appetite	—	1	0 (0.0%)	1 (100.0%)	0 (0.0%)
Others ^c^	18.0 (15.0–32.8)	4	3 (75.0%)	1 (25.0%)	0 (0.0%)

GIAEs: Gastrointestinal adverse events. IQR: Interquartile range. The patterns are defined as follows. Pattern A: Follow-up was conducted 1 and 2 weeks after the initiation of DPP-4 inhibitors, as specified in the community pharmacist-led intervention program; Pattern B: Follow-up was conducted during the patient’s regular pharmacy visits; Pattern C: Follow-up was conducted voluntarily by community pharmacists at times outside those specified in the community pharmacist-led intervention program. ^a^ The median (interquartile range) was calculated for GIAEs observed in two or more cases. ^b^ The number of GIAEs included duplicates when multiple events occurred in a single patient. ^c^ Includes soft stools (two cases), abdominal bloating (one case), and stomach heaviness (one case).

**Table 5:** Progress summary for patients whose information was provided to physicians.

No.	Sex	Age Group	DPP-4 Inhibitor	Case Description
1	Male	70 s	Teneligliptin	- Symptom: Diarrhea confirmed on day 28 during the third follow-up. - Action taken: Pharmacist informed the physician; suspected drug rash was also reported. - Outcome: Discontinuation of teneligliptin confirmed on day 83; diarrhea resolved.
2	Male	80 s	Sitagliptin	- Symptom: Constipation and loss of appetite were identified on day 85 during the second follow-up. - Action taken: Pharmacist informed the physician. - Outcome: Appetite improved by day 123; constipation resolved by day 185; sitagliptin continued.
3	Female	60 s	Linagliptin	- Symptom: Constipation identified on day 4 during the first follow-up. - Action taken: Pharmacist reported symptoms and requested physician confirmation. - Outcome: Constipation resolved by day 12; linagliptin continued up to day 287.
4	Female	70 s	Linagliptin	- Symptom: Constipation reported on day 12; bloating was later noted. - Action taken: Pharmacist informed the physician; patient had skipped doses. - Outcome: Discontinuation of DPP-4 inhibitor due to nausea was confirmed on day 140. visited the pharmacy, and discontinuation of DPP-4 inhibitors owing to nausea was confirmed.
5	Male	60 s	Sitagliptin	- Symptom: Soft stools and constipation were identified on day 18. - Action taken: Ongoing symptoms were reported to the physician on day 87; butyric acid bacteria preparation was prescribed. - Outcome: Symptoms were resolved by day 114.

DPP-4 inhibitors: dipeptidyl peptidase-4 inhibitors.

## Data Availability

The data presented in this study are available on request from the corresponding author due to privacy concerns and the need to protect personal information.

## Supplementary Materials

The following supporting information can be downloaded at: https://www.mdpi.com/article/10.3390/pharmacy13050119/s1, Table S1: List of Onset Timing of Gastrointestinal Adverse Events; Figure S1: Follow-up care checklist.

## Abbreviations

The following abbreviations are used in this manuscript:

DPP-4	Dipeptidyl Peptidase-4
GIAE	Gastrointestinal Adverse Event
GIP	Glucose-dependent Insulinotropic Polypeptide
GLP-1	Glucagon-like Peptide-1
JADER	Japanese Adverse Drug Event Report database
IQR	Interquartile Range
