# Supplementary material for: Evaluation of a Community Pharmacist-Led Intervention Program for Early Detection of Gastrointestinal Adverse Events of Dipeptidyl Peptidase-4 Inhibitors: A Multicenter, Non-Randomized Comparative Study
Source: Pharmacy (Basel). 2025 Aug 28;13(5):119. doi: 10.3390/pharmacy13050119 (PMC12452381; doi:10.3390/pharmacy13050119)
Supplement: Supplementary file 1 [file pharmacy-13-00119-s001.zip › Figure S1_Pharmacy_Funabashi_20250604.pdf]

Figure S1: Follow-up care checklist

No	checklist	Response
1	Presence of gastrointestinal adverse events	Yes ・ No
2	Enter gastrointestinal adverse events	1. Nausea/vomiting<br>2. Anorexia<br>3. Constipation<br>4. Diarrhea<br>5. Other ( )
3	Detailed symptom information	
	Location	
	Quality	
	Quantity	
	Timing	
	Setting	
	Factor	
	Associated manifestation	
4	Progress (if gastrointestinal adverse events were observed previously)	1. Improved<br>2. Slightly improved<br>3. No change<br>4. Slightly worsening<br>5. Worsening
5	Other symptoms not related to gastrointestinal adverse events	
6	Patient instructions	1. Seek immediate medical attention<br>2. Seek medical attention if symptoms persist or worsen<br>3. Inform your doctor at the next visit<br>4. Other ( )
7	Suggestions from community pharmacists to physicians	1. Suggested reduction or discontinuation of medication<br>2. Suggested addition of a symptomatic medication (name of medication:)<br>3. Other ( )
