# Supplementary material for: Evaluation of a Community Pharmacist-Led Intervention Program for Early Detection of Gastrointestinal Adverse Events of Dipeptidyl Peptidase-4 Inhibitors: A Multicenter, Non-Randomized Comparative Study
Source: Pharmacy (Basel). 2025 Aug 28;13(5):119. doi: 10.3390/pharmacy13050119 (PMC12452381; doi:10.3390/pharmacy13050119)
Supplement: Supplementary file 1 [file pharmacy-13-00119-s001.zip › Table S1_Pharmacy_Funabashi_20250604.pdf]

Table S1: List of Onset Timing of Gastrointestinal Adverse Events

GIAEs	Median Days (IQR)	WSP β (95% CI)
Nausea and Vomiting	7.0 (3.0–43.0)	0.55 (0.36-0.78)
Diarrhea	20.0 (5.0–221.0)	0.47 (0.28-0.67)
Constipation	97.0 (21.0–365.0)	0.54 (0.30-0.78)
Loss of appetite	92.0 (15.0–683.0)	0.57 (0.38-0.72)

GIAEs: Gastrointestinal adverse events; IQR, Interquartile range; WSP, Weibull sharp parameter; CI, Confidence interval. The figures in the table are based on data from the JADER database, which contains reports of adverse drug reactions in Japan.
